# Asiatic Acid, Extracted from *Centella asiatica* and Induces Apoptosis Pathway through the Phosphorylation p38 Mitogen-Activated Protein Kinase in Cisplatin-Resistant Nasopharyngeal Carcinoma Cells

**DOI:** 10.3390/biom10020184

**Published:** 2020-01-25

**Authors:** Yen-Tze Liu, Yi-Ching Chuang, Yu-Sheng Lo, Chia-Chieh Lin, Yi-Ting Hsi, Ming-Ju Hsieh, Mu-Kuan Chen

**Affiliations:** 1 Institute of Medicine, Chung Shan Medical University, Taichung 402, Taiwan; 144084@cch.org.tw; 2 Department of Family Medicine, Changhua Christian Hospital, Changhua 500, Taiwan; 3 Department of Holistic Wellness, Mingdao University, Changhua 52345, Taiwan; 4 Oral Cancer Research Center, Changhua Christian Hospital, Changhua 500, Taiwan; 177267@cch.org.tw (Y.-C.C.); 165304@cch.org.tw (Y.-S.L.); 181327@cch.org.tw (C.-C.L.); 180082@cch.org.tw (Y.-T.H.); 5 Graduate Institute of Biomedical Sciences, China Medical University, Taichung 404, Taiwan; 6 Department of Otorhinolaryngology, Head and Neck Surgery, Changhua Christian Hospital, Changhua 500, Taiwan

**Keywords:** Asiatic acid, nasopharyngeal cancer, apoptosis, cisplatin resistance, MAPK pathway

## Abstract

Nasopharyngeal carcinoma (NPC) is an important issue in Asia because of its unique geographical and ethnic distribution. Cisplatin-based regimens are commonly the first-line used chemotherapy, but resistance and toxicities remain a problem. Therefore, the use of anticancer agents derived from natural products may be a solution. Asiatic acid (AA), extracted from *Centella asiatica*, was found to have anticancer activity in various cancers. The aim of this study is to examine the cytotoxic effect and mediated mechanism of AA in cisplatin-resistant NPC cells. The results shows that AA significantly reduce the cell viability of cisplatin-resistant NPC cell lines (cis NPC-039 and cis NPC-BM) in dose and time dependent manners caused by apoptosis through the both intrinsic and extrinsic apoptotic pathways, including altered mitochondrial membrane potential, activated death receptors, increased Bax expression, and upregulated caspase 3, 8, and 9. The Western blot analysis of AA-treated cell lines reveals that the phosphorylation of MAPK pathway proteins is involved. Further, the results of adding inhibitors of these proteins indicates that the phosphorylation of p38 are the key mediators in AA-induced apoptosis in cisplatin-resistant human NPC cells. This is the first study to demonstrate the AA-induced apoptotic pathway through the phosphorylation p38 in human cisplatin-resistant nasopharyngeal carcinoma. AA is expected to be another therapeutic option for cisplatin-resistant NPC because of the promising anti-cancer effect and fewer toxic properties.

## 1. Introduction

Nasopharyngeal carcinoma (NPC) is an important and widely studied issue in Asia, especially in Eastern Asia and South-Eastern Asia, because of its unique geographical distribution. According to the GLOBOCAN estimates in 2018, the estimated number of new cases is 129,079 worldwide, of which 109,221 are in Asia, accounting for 84.6% [1]. Etiological factors for NPC include Epstein–Barr virus (EBV) infection, human papillomavirus (HPV) infection, genetic susceptibility, and salted fish consumption [2]. Different from other head and neck cancer, the primary and only curative treatment for NPC is radiotherapy instead of surgery for early-stage disease. Chemotherapeutic agents are used as concurrent chemoradiotherapy (CCRT), adjuvant chemotherapy, or induction chemotherapy in locoregionally advanced disease and metastatic disease [3]. Cisplatin-based regimen is commonly the first-line used. Paiar el al. proposed that the overall survival (OS) and disease-free survival by CCRT had statistically significant improvement with five-year OS of about 70% in non-metastatic stage III and IV disease, but the efficacy of chemotherapy varied markedly among different trials [4]. Otherwise, chemoradiotherapy leads to various side effects including hematological acute toxicity, mucositis, xerostomia, loss of taste, dysphagia, skin damage, skull bone damage, sensorineural hearing loss, and osteoradionecrosis. Therefore, new effective treatments with fewer toxicities need to be identified. 

Finding anti-cancer agents from natural compound is a burgeoning research filed recent years due to its fewer toxic characteristics. Asiatic acid (AA) is a pentacyclic triterpenoid, one of the subclasses of phytochemicals, and derived from *Centella asiatica* which is a kind of tropical plant used as a diet and medicine [5]. AA has been demonstrated to have antioxidant and anti-inflammatory activity, and to have been involved in numerous molecular mechanisms and cell signaling pathway which related to its cardio-protective, neuro-protective, anti-diabetic, anti-obesity, and anti-cancer effect [5]. Previous studies have reported many mechanisms observed in carcinogenesis and metastatic process. Hsu el al. discovered the anticancer effect of AA in breast cancer through cell cycle arrest and mitochondrial apoptotic pathway [6]. Another research proposed that AA inhibit transforming growth factor-beta1 (TGF-beta1)-induced epithelial-mesenchymal transition in lung cancer [7]. However, the effect of AA on nasopharyngeal cancer cells is still unclear. 

As described above, radiotherapy and chemotherapy are effective in early-stage NPC. In contrast, the median overall survival in distant metastases is only about 12 to 15 months after platinum-based chemotherapy [8]. Multidrug combinations can improve the therapeutic effects, but accompanied with significant hematological and mucosal toxicities, even septic deaths [9]. Even patients who initially respond to cisplatin will recur, and it is often refractory to further platinum therapy [10]. Whether it is innate or acquired resistance, to discover effective and less-toxic agents for anti-cancer or chemosensitizers is important. This study aims to examine the cytotoxic effect and mechanisms of AA on cisplatin-resistance NPC cells.

## 2. Materials and Methods 

### 2.1. Chemicals

Asiatic acid is a triterpenoid isolated from *Centella asiatica* (≥98% purity), purchased from Chemfaces. Molecular Weight: 488.7, Formula: C_30_H_48_O_5_, dissolved in dimethyl sulfoxide (DMSO), and stored at −20 °C. All experiment treatments were consistently less than 0.1% (*v*/*v*) DMSO concentration. Other chemicals were obtained from the following companies: MTT (3-(4,5-dimethylthiazol-2-yl)-2,5-diphenyltetrazolium bromide) and DAPI dye (Sigma Aldrich, St Louis, MO, USA), cisplatin (Enzo Life Sciences), specific inhibitors (Santa Cruz Biotechnology, Santa Cruz, CA, USA).

### 2.2. Cell Culture

The cisplatin-resistant nasopharyngeal carcinoma cell lines (cis NPC-BM and cis NPC-039) were established by human nasopharyngeal carcinoma (NPC) cell lines (NPC-BM and NPC-039), a gift from Dr. Jen-Tsun Lin, Hematology & Oncology, Changhua Christian Hospital, continuous culture with increase concentrations of cisplatin 1–60 nM for 6 months. The culture medium contains 60 nM cisplatin to maintain drug resistance. All cells were cultured in RPMI 1640 medium (with 10% (*v*/*v*) fetal bovine serum (FBS), 1 mM glutamine, 1% (*v*/*v*) penicillin/streptomycin, 1.5 g/l sodium bicarbonate, and 1 mM sodium pyruvate). All cells were cultured at 37 °C in a 5% CO_2_ culture room.

### 2.3. Cell Cytotoxicity Assay

The effect of Asiatic Acid on cell growth was assayed by the MTT (3-(4,5-dimethylthiazol-2-yl)-2,5-diphenyl tetrazolium bromide) method. Briefly, cells were cultured in 96-well plates (1 × 10^4^/well) and treated with various concentrations of AA (0, 25, 50, 75 μM). After the medium was removed, MTT reagent was added to each well (0.5 mg/mL final concentration) with a further incubation for 4 h at 37 °C in 5% (*v*/*v*) CO_2_. After removing the supernatant, DMSO was added to dissolve the formed blue formazan crystals. The absorbance of the converted dye was measured at 595 nm by the ELISA plate reader. Each condition was performed in triplicate and data were obtained from at least 3 separate experiments.

### 2.4. Cell Cycle Analysis

Cells were seeded (5 × 10^5^/well) on 6-well plates overnight and incubated with various concentrations of AA for 24 h. After wards, cells were fixed 70% (*v*/*v*) ice-cold ethanol overnight, and then stained with PI buffer (4 mg/mL PI, 1% (*v*/*v*) Triton X-100, 0.5 mg/mL RNase A in PBS) for 30 min in the dark at room temperature and then filtered through a 40-mm nylon filter (Falcon, Billerica, MA, USA). The cell cycle distribution was analyzed by flow cytometry.

### 2.5. DAPI Staining

Observation of morphology by DAPI staining cis NPC-BM and cis NPC-039 cells treated with different concentrations of AA (0, 25, 50, and 75 μM) were incubated for 24 h. The cells were fixed with 4% (*v*/*v*) formaldehyde for 30 min at room temperature and permeabilized with 0.1% (*v*/*v*) Triton X-100. Washed twice in PBS, cells were stained with DAPI (50 μg/mL) for 15 min in the dark, then after being washed in PBS, morphological changes were photographed using fluorescence microscopy (Lecia, Bensheim, Germany). Quantitatively, the percentage of apoptotic cells by number of dots was scored on at least 500 cells.

### 2.6. Annexin V/PI Double Staining Assay

Cells were seeded on 6-well overnight and then treated with different concentrations of AA for 24 h. Cells were harvested and suspended in PBS (2% (*v*/*v*) BSA), and then incubated with Muse™ Annexin V & Dead Cell reagent (EMD Millipore, Billerica, MA, USA) for 20 min at room temperature in dark. Samples were analyzed by Muse Cell Analyzer flow cytometry (EMD Millipore, Billerica, MA, USA) and by MUSE 1.4 Analysis software (EMD Millipore).

### 2.7. Mitochondrial Membrane Potential Measurement

Cells were seeded on 6-well plates and incubated with different dose of AA for 24 h. The cells were harvested, washed and suspended in Muse MitoPotential working solution at 37 °C for 20 min. Add 5 μL of 7-AAD and incubate at room temperature for 5 min. Samples were monitored by a Muse Cell Analyzer flow cytometry (EMD Millipore) The data were analyzed by a Muse Cell Analyzer (Millipore).

### 2.8. Western Blotting Analysis

The cells are washed twice with cold PBS and lysed with RIPA buffer containing protease inhibitor cocktail and phosphatase inhibitor cocktail. The protein quantification of supernatants was determined using BCA protein assay (Pierce) and all samples are separated by sodium dodecyl sulfate polyacrylamide gel electrophoresis (SDS-PAGE) and the separated proteins were transferred electrophoretic ally from the gel to the surface of PVDF membrane (Millipore, Bedford, MA, USA). Membranes were blocked with 5% non-fat milk in TBST for 1 h. The analysis used primary antibodies as described by the manufacturers of the antibodies. Antibodies were purchased from Cell Signaling Technology, Inc. (Danvers, MA, USA). Anti-ERK 1/2 (#4695), Anti-JNK 1/2 (#9252), Anti-p38 (#8690), Anti-Akt (#4685), Anti-phospho-ERK 1/2 (#4370), Anti-phospho-JNK 1/2 (#4668), Anti-phospho-p38 (#4511), Anti-phospho-Akt (#4060), Anti-cleaved caspase 3 (#9664), Anti-cleaved caspase 8(#9496), Anti-cleaved caspase 9 (#9505), Anti-cleaved PARP(#5625), Anti-Bax (#2772), Anti-Bak (#3814), Anti-Fas (#4233), Anti-TNF-R1(#3736), Anti-DCR2 (#8049), Anti-DR5 (#8074) or isotype-control rabbit mAbs (Cell Signaling Technology, Beverly, MA). After the final rinsing with TBST, the membrane was incubated with secondary HRP-linked anti-rabbit IgG or anti-mouse IgG for 1 h. After washing, the immunoblots were visualized using chemiluminescence (ECL) HRP substrate (Millipore).

### 2.9. Statistical Analysis

Values represent the means ± standard deviation and the experiments were repeated at least three times. Data were analyzed using Student’s t-test when two groups were compared. A *p* value of <0.05 was considered statistically significant. Statistical analysis using one-way analysis of variance (ANOVA). Analysis of more than three groups using Dunnett’s test.

## 3. Results

### 3.1. Cytotoxicity of Asiatic acid on Cisplatin-Resistance Human NPC Cell Lines

**Figure 1:**
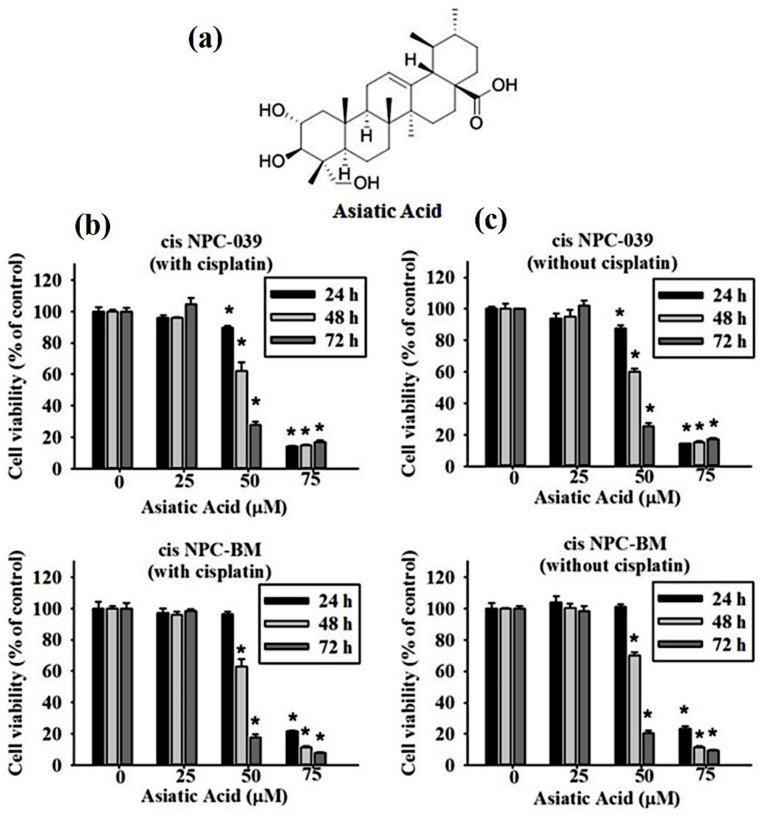
Cytotoxic effects of Asiatic acid on cisplatin-resistant human NPC cell lines (NPC-039 and NPC-BM) (**a**) The chemical structure of Asiatic acid. (**b**) The cell viability significantly decreased in AA-treated cis NPC-BM cell and cis NPC-039 cell with cisplatin and (**c**) without cisplatin in a dose and time dependent manner under MTT assay. * *p* < 0.05 as compared with control group.

Figure 1a reveals the chemical structure of Asiatic acid, a pentacyclic triterpene, titrated extract from *Centella asiatica*. We assessed the cell viability of two kinds of cisplatin-resistant human NPC cell lines, cis NPC-039 and cis NPC-BM, by MTT assay. These cell lines were treated with an increasing concentration of AA (0, 25, 50, and 75 μM) with or without cisplatin for 24, 48, and 72 h. The results in Figure 1b,c show that the cell viability of both cis NPC-039 and cis NPC-BM was inhibited in a dose- and time-dependent manner regardless of whether with or without cisplatin. Cell viability was significantly reduced in concentration of 50 and 75 μM compared to control (0 μM).

### 3.2. Asiatic Acid Induced Cell Apoptosis and Cell Cycle Arrest in Cisplatin-Resistance Human NPC Cell Lines

**Figure 2:**
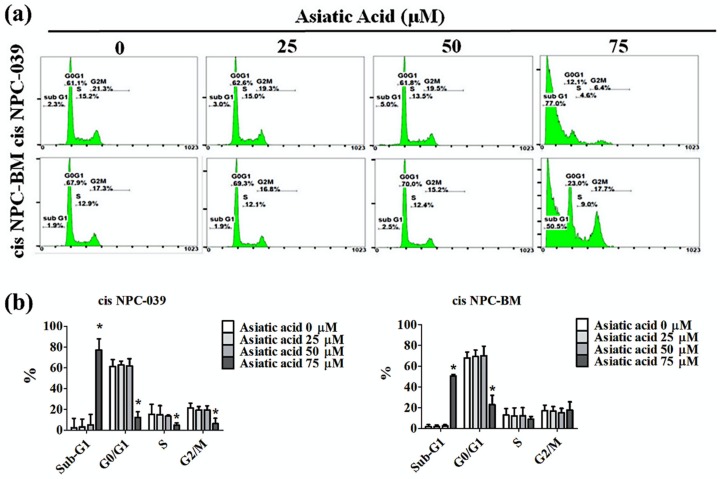
Asiatic acid cause cell cycle arrest in cis NPC-039 and cis NPC-BM cell lines. (**a**) The cisplatin-resistant human NPC cell lines were treated with Asiatic acid (0, 25, 50, and 75 μM). Cell cycle phase distribution (Sub-G1, G0/G1, S, and G2/M) were estimated by flow cytometry. (**b**) The rate of different phase of cell cycle in cis NPC-039 and cis NPC-BM cell lines. * *p* < 0.05 as compared with control group.

**Figure 3:**
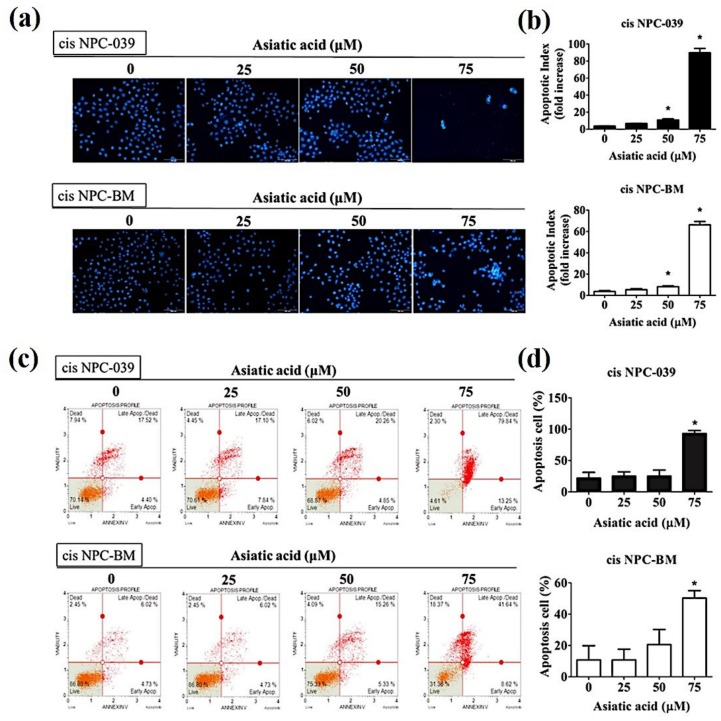
Effect of Asiatic acid on apoptosis induction in cis NPC-039 and cis NPC-BM cell lines. (**a**) Nuclear counterstain level was detected by DAPI staining. Characteristic ‘blebbing’ morphology of cell nucleus was observed by fluorescence microscopy. (**b**) The DNA condensation fold compared with control. (**c**) and (**d**) Cell apoptosis was analyzed by flow cytometric with Annexin-V/PI Staining. * *p* < 0.05 as compared with control group.

PI staining and flow cytometry were applied to determine the cell cycle distribution of cisplatin-resistance human NPC cell lines, cis NPC-039 and cis NPC-BM, and to examine the effect of AA on cell growth suppression. Figure 2 shows that the proportion in the sub-G1 phase increased in a concentration-dependent manner, especially in 75 μM group. Figure 3a illustrates the more condensed nuclei, suggesting the typical characteristic morphology of DNA fragmentation in apoptotic cells, in the higher concentration group of AA-treated NPC cell lines with the fluorescence microscopy after DAPI staining. The difference was statistically significant in 50 and 75 μM groups of both two cell lines (Figure 3b). Furthermore, we performed the Annexin-V and PI double staining and followed by flow cytometric analysis. The results in Figure 3c,d indicated that AA increased apoptotic cells in early stage and late stage of apoptosis, and significantly in 75 μM -treated cell lines.

### 3.3. Asiatic Acid Induced Cell Apoptosis in Cisplatin-Resistance Human NPC Cell Lines through the Mitochondrial Pathway and Death Receptor Related Pathway

**Figure 4:**
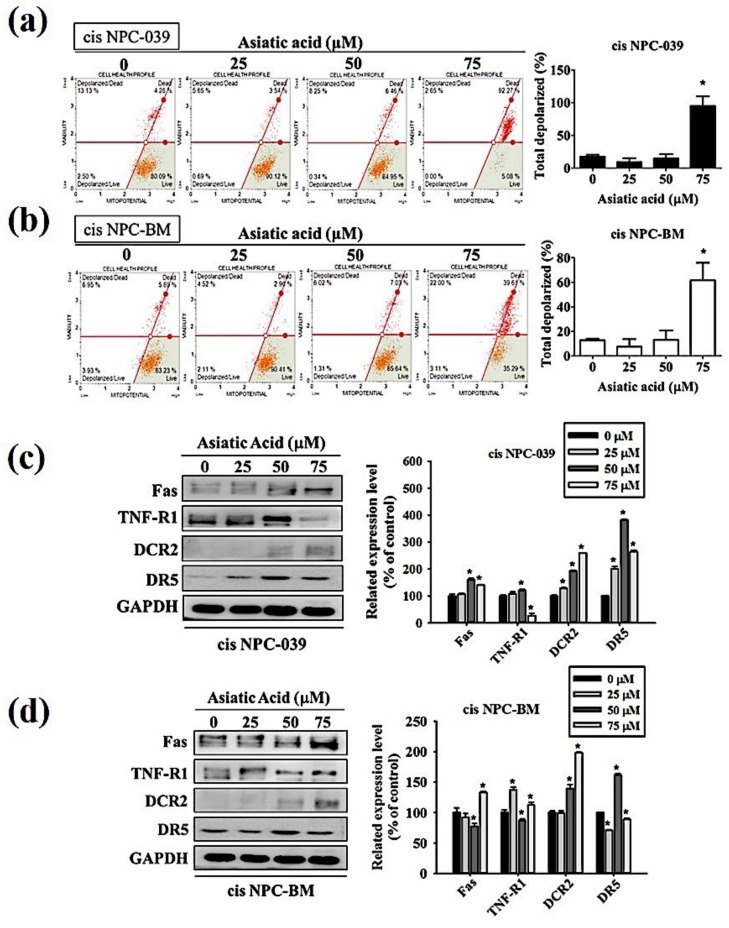
Asiatic acid cause cis NPC-039 and cis NPC-BM cell apoptosis thought the intrinsic pathway and the extrinsic pathway. (**a**) Effect of Asiatic acid on mitochondrial membrane potential was analyzed by flow cytometric (**b**) The related mitochondrial membrane depolarized levels compared with control. (**c**) The expression levels of Fas, TNF-R1, DcR2, and DR5 were detected by Western blot. (**d**) The relative band density was normalized to GAPDH as a densitometer. * *p* < 0.05 versus control group.

The pathway involved in the AA-induced apoptosis in cis NPC-039 and cis NPC-BM cell lines have to be verified. We performed the Muse MitoPotential Kit and Muse Cell Analyzer assays. The results in Figure 4a,b display that the mitochondrial membrane potential was changed and the depolarized cells was significantly increased with AA at 75 μM. On the other hand, the expressions of Fas, DCR2, and DR5 were all increased in AA-treated cisplatin-resistant NPC cell lines by Western blot analysis (Figure 4c,d).

### 3.4. Asiatic Acid Upregulates the Expression of Caspase-3, -8, -9, and Altered the Expression of Bax and Bak in Cisplatin-Resistance Human NPC-039 and NPC-BM Cell Lines

**Figure 5:**
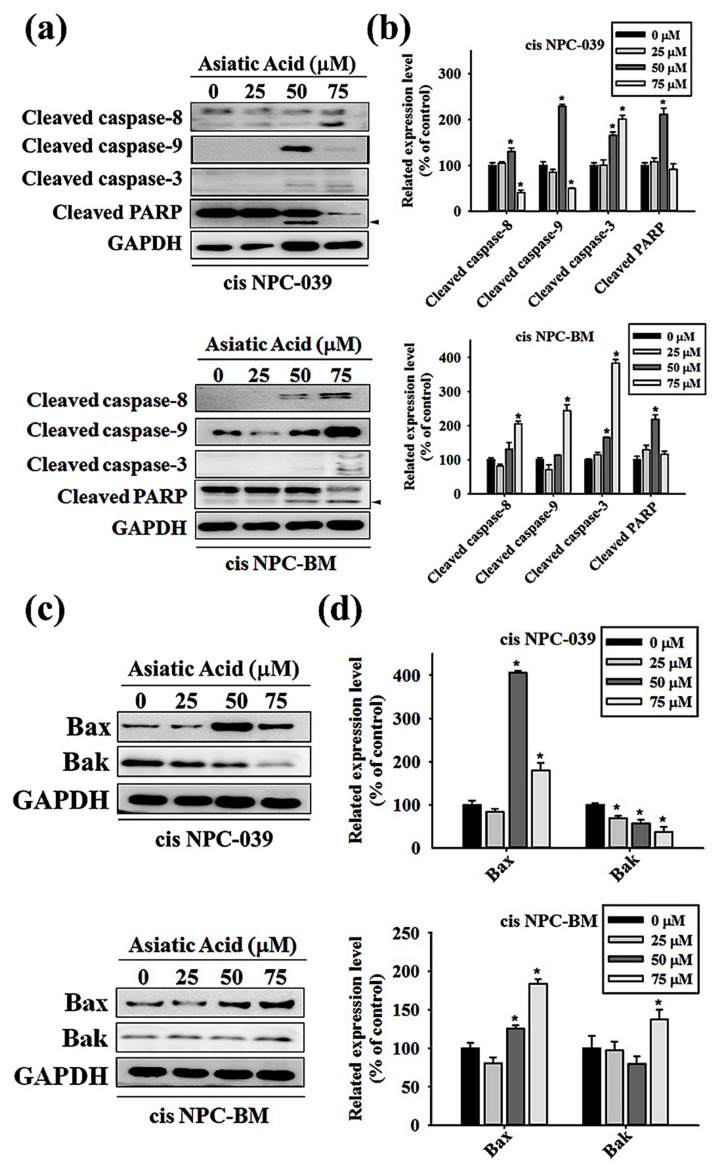
Asiatic acid activates caspase 3, 8, and 9 and upregulated the expression of Bax and Bak. (**a**) The expression levels of cleaved caspase-3, -8, -9 and PARP were detected by Western blot. (**b**) The protein levels were quantified and normalized to GAPDH using a densitometer. (**c**) The expression levels of Bak and Bak were detected by Western blot. (**d**) The protein levels were quantified and normalized to GAPDH using a densitometer. * *p* < 0.05 versus control group.

We performed Western blot analysis of the cleaved forms of caspase-3, caspase-8, caspase-9, and Poly (ADP-ribose) polymerase (PARP) in AA-treated NPC cell lines to determine the caspase activation pathway in AA-induced apoptosis. As observed in Figure 5a,b, the expressions of caspase-3, -8, -9, and PARP increase significantly in cis NPC-039 cell lines with AA at 50 μM compared to control group. In cis NPC-BM cell clines, significantly increased expression of caspase-3, -8, and -9 was found in AA treatment of 75 μM for 24 h. In Figure 5c,d, the related expression level of Bax and Bak, two pro-apoptotic proteins, in cis NPC-BM cell line were significantly increased by 183% and 137% respectively. In another cis NPC-039 cell line, 24-h 50 μM and 75 μM AA treatment increased Bax expression, but decreased Bak expression.

### 3.5. Asiatic Acid Induced Cell Apoptosis via p38 Pathway in Cisplatin-Resistance Human NPC-039 and NPC-BM Cell Lines

**Figure 6:**
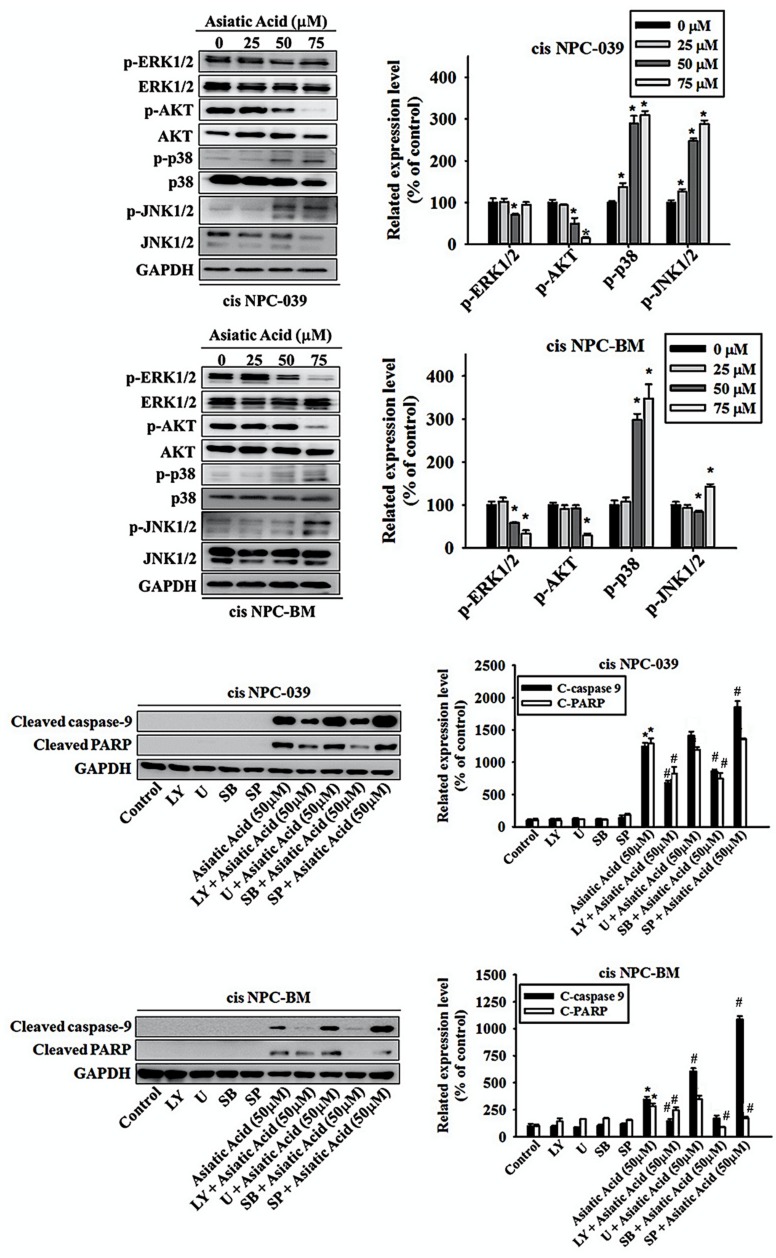
Effect of Asiatic acid on the MAPKs and AKT pathway in cis NPC-039 and cis NPC-BM cell lines (**a**) The expression levels of ERK1/2, AKT, p38, and JNK1/2 were detected by Western blot. (**b**) The protein levels were quantified and normalized to GAPDH using a densitometer. * *p* < 0.05 versus control group. (**c**) After pretreated with ERK1/2, AKT, p38, and JNK1/2 inhibitors separately (U, LY, SB, SP), the expression levels of cleaved caspase-9 and PARP were detected by Western blot. (**d**) The protein levels were quantified and normalized to GAPDH using a densitometer. * *p* < 0.05 versus control group. # *p* < 0.05, compared with Asiatic acid treated only.

We applied the Western blot analysis to obtain the level of expression of ERK1/2, AKT, p38, and JNK1/2 to confirm the mechanisms related to AA-mediated apoptosis. The results in Figure 6a,b display the significantly increased expressions of phosphorylation of p38 and JNK1/2 with dose-dependent relationship in cis NPC-039 cell lines, whereas decreased the phosphorylation of AKT in 50- and 75 μM -treated groups and ERK1/2 in 50 μM-treated group. In cis BPC-BM cell lines, AA treatment of 50 and 75 μM for 24 h significantly increased the activation of phosphor-p38 by 298% and 348% respectively and significantly decreased the expression of phosphor-ERK1/2. Decreased expression of phosphor-AKT and increased of phosphor-JNK1/2 were found in 75 μM-treated cell group. To further examine the signal transduction pathways involved in apoptosis, we treated cis NPC-039 and cis NPC-BM cell lines with AA of 50 μM plus ERK1/2 inhibitor (U), AKT inhibitor (LY), p38 inhibitor (SB), and JNK1/2 inhibitor (SP). The related expression level of cleaved caspase 9 was increased when adding ERK1/2 inhibitor (U) and JNK1/2 inhibitor (SP) and decreased when adding AKT inhibitor (LY) and p38 inhibitor (SB) in both cell lines compared to AA-treated group. The expression of cleaved PARP was increased when adding JNK1/2 inhibitor (SP) and decreased when adding AKT inhibitor (LY), and p38 inhibitor (SB) in cis NPC-039. However, the expression of cleaved PARP was increased when adding ERK1/2 inhibitor (U) and decreased when adding AKT inhibitor (LY), p38 inhibitor (SB), and JNK1/2 inhibitor (SP) in cis NPC-BM. Therefore, we might propose that the phosphorylation of p38 plays a considerable role in the pathway of AA-related apoptosis. 

## 4. Discussion

Chemotherapy is one of the mainstay options for the treatment against nasopharyngeal carcinoma, especially in the locoregionally advanced or metastatic stage. Cisplatin is most commonly used, but the therapeutic outcome may be restricted because of its toxicity and resistance. This research attempted to discover the promising natural compound against cisplatin-resistance NPC cells. Our results indicate that the Asiatic acid may induce apoptosis in cisplatin-resistance NPC-039 and NPC-BM cell lines via the p38 and ERK signaling transduction pathway.

It has been proposed that Asiatic acid has various pharmacological activities such as anti-inflammatory and antioxidant, as well as the potent anti-hypertensive, neuro and cardio-protective, antimicrobial, and antitumor activities [5]. Many studies had proved the anti-cancer effects in vitro or in vivo of AA in breast cancer, ovary cancer, colon cancer, gastric cancer, cholangiocarcinoma, glioblastoma, lung cancer, lymphoma, and melanoma [6,11,12,13,14,15,16,17,18]. Our study corresponds with these researches and revealed the anti-cancer effect of AA in both dose and time dependent manners in cisplatin-resistance NPC cell lines (Figure 1b). AA induced cell death by cell cycle arrest and apoptosis in this study (Figure 2 and Figure 3). Previous study also revealed that AA induced cells undergo S-G2/M phase arrest and apoptosis in human breast cancer MCF-7 and MDA-MB-231 cell lines [6]. Siddique et al. suggested that AA induced apoptosis through decreasing the Bcl-2 and increasing the Bax, caspase-3 and -9 in a rat model of colon carcinogenesis [11]. 

Furthermore, we investigated the intrinsic and extrinsic pathway in apoptosis induced by AA. Figure 4 shows that both the mitochondrial pathway and the death receptor-initiated pathway are involved in AA-induced apoptosis, as well as the upregulated Bax and Bak in Figure 5. The research in 2017 revealed that AA-induced apoptosis in lung cancer was caused by altering the mitochondrial membrane potential and lead to activation of caspase and PARP signaling pathways [14]. Related researches mention that AA cause activation of mitochondrial pathway via regulating B cell CLL/lymphoma 2 (BCL2) family proteins and increasing the ratio of Bax to Bcl2 which lead to cancer cell apoptosis [19,20]. 

Previous studies revealed that the AA-induced apoptosis of human cancer cell is mediated by the mitogen-activated protein kinase (MAPK) pathway with or without involving the activated caspases [5,6,21]. The inhibition of ERK and p38 phosphorylation in AA-treated cancer cell are also noted [15]. Our results in Figure 5 and Figure 6 also indicate that caspase-3, -8, and -9 are upregulated in AA-induced apoptosis and that caspase-9 expression is mediated by the phosphorylation of p38 and ERK1/2 pathway in human NPC cell lines. Other research has indicated the anti-cancer activity of AA by Akt pathway [17,22], but our results didn’t show it.

Based on previous research, the resistance of cisplatin comes from three mechanisms: altered DNA repair, cytosolic inactivation of drug, and altered cellular accumulation of drug, and the increased expression of excision repair cross complementation 1 (ERCC1) to alter DNA repair in NPC was related to cisplatin resistance [10]. Liu et al. proposed that the resistance might be sensitized by increasing Bax/Bcl-2 mRNA ratios caused by inhibited NFκB expression and downregulating ERCC1 and thymidine phosphorylase (TP) by inhibiting the phosphorylation of ERK, p38 and PI3K/AKT pathways, which all lead to apoptosis, in human hepatoma HepG2 cells [23]. Another research also revealed the decreased ERCC1 mRNA expression by downregulation of p38 MAPK pathway [21]. Consistent with these studies, our results revealed significantly increased expression of Bax and decreased phosphorylation of ERK in AA-treated cis-NPC cell lines. However, the phosphorylation of p38 was upregulated in our study. Hsieh et al. also proposed that another plant-derived triterpene, Celastrol, also induces cancer cell apoptosis of cisplatin-resistant nasopharyngeal carcinoma though increasing p38 MAPK signaling pathway, as well as ERK1/2 [24]. These inconsistent results of the regulatory signaling pathway and detailed mechanisms require further clarification.

## 5. Conclusions

In conclusion, this is the first study to demonstrate the AA-induced apoptotic pathway in human cisplatin-resistant nasopharyngeal carcinoma. The mechanism of both intrinsic and extrinsic pathways, including the phosphorylation p38 and Bax upregulation, is involved. Therefore, AA is expected to be another therapeutic option for cisplatin-resistant NPC because of the promising anti-cancer effect and fewer toxic properties. 

## Data Availability Statement

All the data that supports the findings of this study are available in this article.

## Abbreviations

Nasopharyngeal carcinoma (NPC); Asiatic acid AA); Extracellular signal–regulated kinases (ERKs); c-Jun N-terminal kinases (JNKs); Mitogen-activated protein kinases (MAPK); Propidium iodide (PI)
